# Impact of MicroRNAs in Interaction With Environmental Factors on Autism Spectrum Disorder: An Exploratory Pilot Study

**DOI:** 10.3389/fpsyt.2021.715481

**Published:** 2021-10-05

**Authors:** LiHua Cui, WenRan Du, Ning Xu, JingYi Dong, BingJie Xia, JingYi Ma, RuoTong Yan, LanYing Wang, FuMin Feng

**Affiliations:** ^1^School of Public Health, North China University of Science and Technology, Tangshan, China; ^2^Department of Child Health Care, Tangshan Maternal and Child Health Care Hospital, Tangshan, China; ^3^Department of Child Health Care, Fengrun District Maternal and Child Health Care Hospital of Tangshan, Tangshan, China

**Keywords:** autism spectrum disorder, microRNA, environment, risk, etiology

## Abstract

**Background:** This study aimed to explore the main effects of environmental risk factors as well as their interaction effects with miRNA on the risk of autism spectrum disorder (ASD).

**Methods:** One hundred fifty-nine ASD children (ASD group) and 159 healthy children (control group), aged 2–6 years, were included in this study. ASD diagnoses were based on DSM-5 criteria. The extensive medical and demographic characterization of the two groups were recorded. MicroRNAs (miRNAs) in serum were detected by qRT-PCR.

**Results:** Compared with the control group, the ASD group had significantly higher rates of maternal stress during pregnancy (*p* < 0.001), maternal drinking during pregnancy (*p* = 0.006), threatened abortion (*p* = 0.011), pregnancy-induced hypertension (*p* = 0.032), gestational diabetes (*p* = 0.039), maternal anemia during pregnancy (*p* < 0.001), umbilical cord knot (*p* < 0.001), neonatal jaundice (*p* < 0.001), family psychiatric history (*p* = 0.001), and much lower birth weight (*p* = 0.012). Furthermore, the ASD group had much lower expression levels of hsa-miR-181b-5p (*p* < 0.001) and hsa-miR-320a (*p* < 0.001) and significantly higher levels of hsa-miR-19b-3p (*p* < 0.001). The interactions of hsa-miR-320a and maternal stress during pregnancy (OR = 39.42, *p* < 0.001), hsa-miR-19b-3p and neonatal jaundice (OR = 2.44, *p* < 0.001), and hsa-miR-181b-5p and family psychiatric history (OR = 8.65, *p* = 0.001) could increase ASD risk.

**Conclusions:** The dysregulation of hsa-miR-181b-5p, hsa-miR-320a, and hsa-miR-19b-3p could interact with environmental factors, such as maternal stress during pregnancy, neonatal jaundice, and family psychiatric history, to impact the risk of ASD.

## Background

As a heterogeneous brain-based neurodevelopmental disorder, autism spectrum disorder (ASD) is characterized by a continuum of deficits in communication, social interaction, behavior, and restricted interests (1, 2). It is reported that the annual incidence of ASD is between 1 and 3% (3). The prevalence of ASD is sex imbalance with a distribution of three males to one female (4, 5). ASD often impairs social skills and autonomy, causing ASD children with difficulty in social, speech, and behavioral skill development.

The etiological factors of ASD remain largely unknown. However, it has been reported that risk factors, such as genetics, environmental factors, prenatal and perinatal factors, are involved in the development of ASD (6). Among these, genetics occupies the main factor. ASD has a complex genetic background (7). Approximately 10% of ASD patients are reported to have an identifiable genetic cause (8). The characteristics of highly genetic heterogeneity made the pathophysiology of ASD really elusive (4). MicroRNAs (miRNAs) are short noncoding RNAs with 18–25 nucleotides, playing an important role in regulating gene expression of ASD patients (9). Previously, miRNAs were known to be essential for normal brain development and function, making them attractive biomarker candidates for central nervous system disorders (2). It is reported that miRNAs are also closely associated with the pathogenesis of ASD (2). MicroRNAs, such as miR-146a, miR-19b, miR-181b, hsa-miR-320a, and miR-107 in brain tissues, serum, and/or saliva, could be used as diagnostic biomarkers of ASD (10).

Environmental factors are also important for the etiology of ASD. According to several previous studies, environmental factors, including drugs, toxic exposures, parental age, nutrition, and fetal environment, make up 40–50% of variance in ASD liability (11). For example, it is revealed by a meta-analysis of 27 studies that parental age is associated with the risk of ASD in children (12). Two meta-analyses focus on the associations between ASD risk and obstetric factors; they find that factors such as umbilical cord complications, maternal hemorrhage, low birth weight, and genital malformation were associated with the risk of ASD (13, 14).

A recent study shows that the interaction of genetic and environmental risk factors could exacerbate ASD symptoms (15), suggesting that gene–environment interaction may be a potential mechanism to reveal the etiology of ASD. At present, few studies focus on the interaction of miRNAs and environmental risk factors in children with ASD. Thus, we conducted this study to explore the main effects of miRNAs as well as their interaction effects with well-replicated ASD environmental risk factors on the risk of ASD.

## Materials and Methods

### Subjects

This multicenter, cross-sectional study included ASD and healthy children, aged 2–6 years. The recruitment occurred between June 2018 and June 2020 at Tangshan Maternal and Child Health Care Hospital and two Special Training Centers. Children were diagnosed as ASD according to the Diagnostic and Statistical Manual of Mental Disorders (DSM-5) criteria (16) assessed by two developmental pediatricians with at least 10 years of experience. The inclusion criteria for ASD children (ASD group) were clinical diagnosis of ASD and the absence of other medical, neurological, genetic, or metabolic condition. Healthy children for the same period in Tangshan Maternal and Child Health Care Hospital child health care department without any history of ASD were enrolled as the control group.

This study was approved by the ethical committee of North China University of Science and Technology, and written informed consent was obtained from the parents.

### Subject Characterization

For all subjects in the two groups, extensive medical and demographic characteristics were collected from existing medical records, including sex, age, paternal/maternal age, maternal stress during pregnancy (the psychosocial stress of pregnant woman), maternal smoking during pregnancy, maternal drinking during pregnancy, toxic exposure during pregnancy, threatened abortion (threatened abortion was diagnosed when vaginal bleeding with or without abdominal pain occurred before 28 weeks of gestation), premature birth (premature birth was defined as the birth occurring after 28 weeks and before 37 weeks of gestation), pregnancy-induced hypertension, gestational diabetes, maternal anemia during pregnancy, multivitamin intake during pregnancy, and family psychiatric history. Autistic traits have been screened with the Childhood Autism Rating Scale (CARS) (17): a (raw) score of ≥30 indicates the probability of an ASD.

### RNA Extraction and Quantitative Real-Time Reverse Transcription PCR

Blood samples were collected from all children in a nonfasting state; 4 mL of peripheral venous blood was collected and centrifuged at 3,000 rpm for 10 min at 4°C to separate the pellet and serum. The serum was stored at −80°C until analysis.

The TRIZOL reagent (Invitrogen, USA) was used to isolate serum total RNA. The miRcute miRNA cDNA First-Strand Synthesis Kit (Tiangen, China) was used to synthesize the cDNA. The expressions of hsa-miR-181b-5p, hsa-miR-19b-3p, and hsa-miR-320a were detected by SYBR^®^ Premix Ex Taq TM II (Takara, Japan). The PCR upstream primers were shown as follows: hsa-miR-181b-5p, 5′-AACAUUCAUUGCUGUCGGUGGGU-3′; hsa-miR-19b-3p, 5′-UGUGCAAAUCCAUGCAAAACUGA-3′; hsa-miR-320a, 5′-AAAAGCUGGGUUGAGAGGGCGA-3′; U6, 5′-GCAAGGATGACACGCCAAT-3′. The downstream primers were mRQ 3′ universal primers provided by the reverse transcription kit. The primers were synthesized by Shanghai Shenggong Bioengineering Co., LTD. The qRT-PCR was run on ABI StepOne Plus (Applied Biosystems, CA, USA) using a two-step PCR protocol: an initial denaturation step at 95°C for 10 min, followed by 40 cycles with a denaturation step at 95°C for 2 min and an annealing/extension step at 60°C for 60 s. The miRNA expression was calculated using the 2^−ΔΔ^Ct method.

### Statistical Analyses

SPSS 22.0 (IBM Corp., NY, USA) was used to analyze the data. Demographic characteristics were compared between the ASD and control groups using *t*-test (two-tailed) for normal distribution continuous variables, Kruskal–Wallis rank test for nonnormal distribution continuous variables, or χ^2^ test for categorical variables when appropriate. The comparison of miRNA expression between the two groups also used two-tailed *t*-tests. Multivariable logistic regression was used to assess possible risk factors for ASD. If the variables exhibited statistically significant contributions shown by the likelihood ratio test, they could be included in the model. *P* < 0.05 were considered statistically significant.

## Results

### Demographic Characteristics

A total of 318 children aged 2–6 years were recruited to this study. Of them, 159 children with ASD were assigned to the ASD group, and the remaining 159 healthy children were enrolled as controls. The demographic characteristics of the ASD and control groups are shown in Table 1. Compared with the control group, the ASD group had much higher scores of CARS (*p* < 0.001), significantly higher rates of maternal stress during pregnancy (*p* < 0.001), maternal drinking during pregnancy (*p* = 0.006), threatened abortion (*p* = 0.011), pregnancy-induced hypertension (*p* = 0.032), gestational diabetes (*p* = 0.039), maternal anemia during pregnancy (*p* < 0.001), umbilical cord knot (*p* < 0.001), neonatal jaundice (*p* < 0.001), family psychiatric history (*p* = 0.001), and much lower birth weight (*p* = 0.012).

**Table 1:** Comparison of demographic characteristics between ASD children and healthy controls.

**Characteristics**	**Control group (*n* = 159)**	**ASD group (*n* = 159)**	***P*-value**	**Odds ratio (OR)**	**Adjusted *P*-value**
Age (years)	3.30 ± 0.78	3.13 ± 0.91	0.077^a^	0.79	0.078
Males/females	137/22	142/17	0.393^b^	1.34	0.438
Childhood Autism Rating Scale [Mean (range)]	12.2 (6–19)	38.6 (24–47)	<0.001^c^	–	–
Paternal age (years)	33.10 ± 6.62	33.18 ± 5.73	0.913^a^	1.00	0.913
Maternal age (years)	31.92 ± 4.87	31.49 ± 5.24	0.452^a^	0.98	0.450
Maternal stress during pregnancy [*n* (%)]	2 (1.26)	48 (30.19)	<0.001^b^	33.95	<0.001
Maternal smoking during pregnancy [*n* (%)]	2 (1.26)	8 (5.03)	0.054^b^	4.16	0.075
Maternal drinking during pregnancy [*n* (%)]	2 (1.26)	12 (7.55)	0.006^b^	6.41	0.016
Toxic exposure during pregnancy [*n* (%)]	5 (3.14)	12 (7.55)	0.081^b^	2.51	0.090
Threatened abortion [*n* (%)]	6 (3.77)	18 (11.32)	0.011^b^	3.26	0.015
Premature birth [*n* (%)]	14 (8.81)	8 (5.03)	0.185^b^	0.55	0.190
Pregnancy-induced hypertension [*n* (%)]	2 (1.26)	9 (5.66)	0.032^b^	4.71	0.034
Gestational diabetes [*n* (%)]	1 (0.63)	6 (3.77)	0.039^b^	6.20	0.023
Maternal anemia during pregnancy [*n* (%)]	12 (7.55)	44 (27.67)	<0.001^b^	4.69	<0.001
Multivitamins intake during pregnancy [*n* (%)]	25 (15.72)	33 (20.75)	0.245 ^b^	1.40	0.247
Cesarean delivery [*n* (%)]	101 (63.52)	96 (60.38)	0.564 ^b^	0.88	0.729
Birth weight (kg)	3.49 ± 0.51	3.35 ± 0.48	0.012^a^	0.56	0.013
Umbilical cord knot [*n* (%)]	20 (12.58)	48 (30.77)	<0.001^b^	3.01	<0.001
Neonatal jaundice [*n* (%)]	31 (19.50)	81 (50.94)	<0.001^b^	4.29	<0.001
Family psychiatric history [*n* (%)]	4 (2.52)	19 (11.95)	0.001^b^	5.26	0.003

a *Student's t-test*;

b *Chi-square tests*;

c *Kruskal–Wallis rank test*.

### The Comparison of miRNAs Expression Between the Two Groups

**Figure 1:**
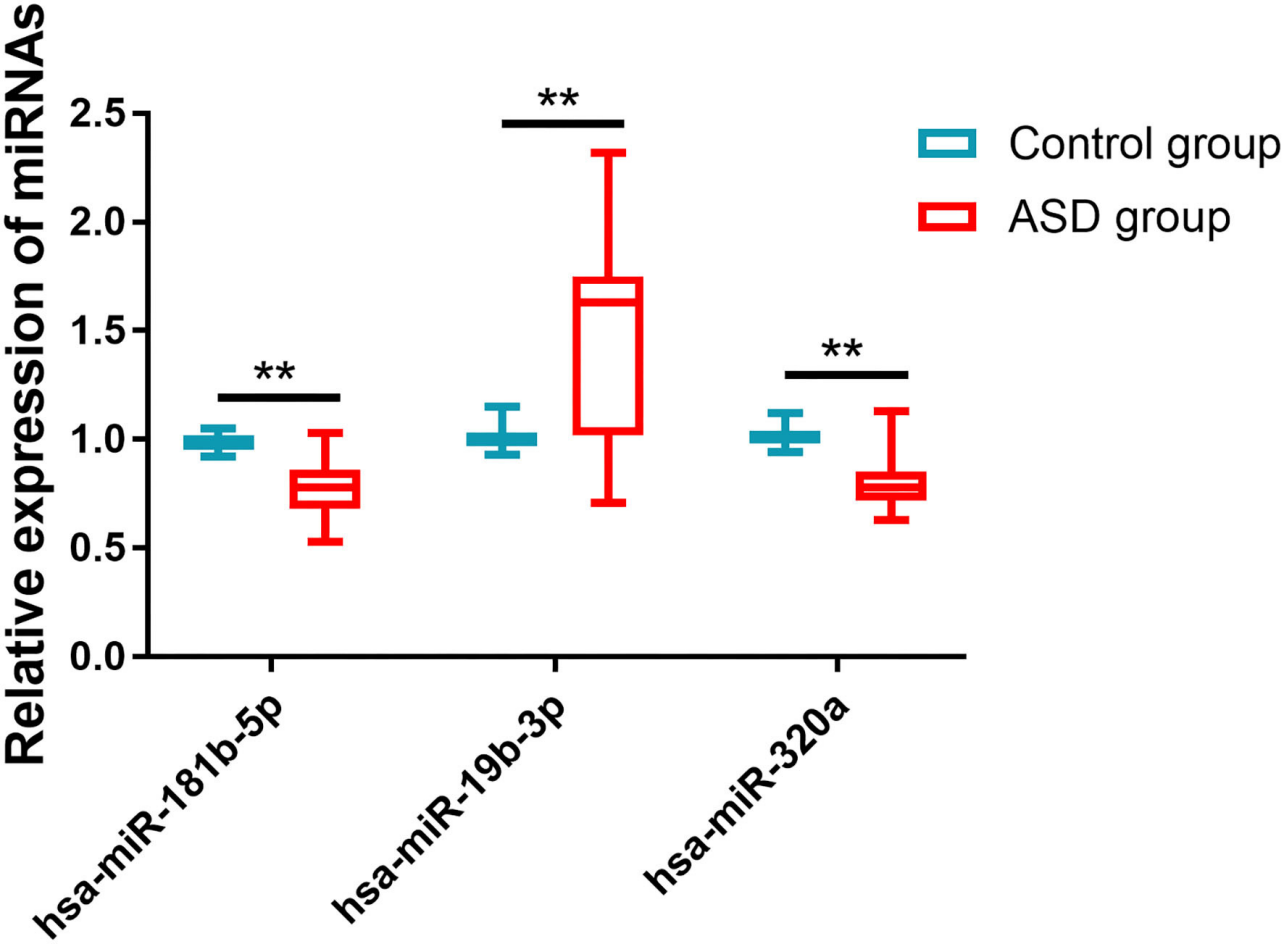
The expressions of miRNAs in two groups. Compared with the control group, the ASD group had much lower expression levels of hsa-miR-181b-5p and hsa-miR-320a and significantly higher levels of hsa-miR-19b-3p. ^**^p < 0.001 vs. control group.

The relative expressions of hsa-miR-181b-5p, hsa-miR-320a, and hsa-miR-19b-3p in the ASD and control groups were detected by qRT-PCR. As shown in Figure 1, the ASD group had much lower expression levels of hsa-miR-181b-5p (0.774 ± 0.131 vs. 0.991 ± 0.037, *p* < 0.001) and hsa-miR-320a (0.805 ± 0.121 vs. 1.010 ± 0.043, *p* < 0.001) and a significantly higher level of hsa-miR-19b-3p (1.502 ± 0.413 vs. 1.004 ± 0.044, *p* < 0.001) compared with the control group.

### The Impact of Environmental Factors and miRNA Interaction on the Risk of ASD

Based on the results of univariate analysis, risk factors, including maternal stress during pregnancy, maternal drinking during pregnancy, threatened abortion, pregnancy-induced hypertension, gestational diabetes, maternal anemia during pregnancy, umbilical cord knot, neonatal jaundice, much lower birth weight and family psychiatric history and their interactions with hsa-miR-181b-5p, hsa-miR-320a, or hsa-miR-19b-3p were included in the multivariable logistic regression model. As shown in Table 2, the expressions of has-miR-181b-5p (OR = 0.002, 95% CI: 0–0.014; *p* < 0.001) and hsa-miR-320a (OR = 0.001, 95% CI: 0–0.005; *p* < 0.001) decreased the ASD risk. Some environmental factors could interact with miRNAs and alter the effect of miRNAs on ASD. Having maternal stress during pregnancy was associated with hsa-miR-320a expression with 39.42-fold increased odds of ASD risk (OR = 39.42, 95% CI: 6.07–255.84; *p* < 0.001), having neonatal jaundice was associated with hsa-miR-19b-3p expression with a 2.44-fold increase (OR = 2.44, 95% CI: 1.55–3.83; *p* < 0.001), and having a family psychiatric history was associated with hsa-miR-181b-5p expression with a 8.65-fold increase (OR = 8.65, 95% CI: 2.23–33.58; *p* = 0.001).

**Table 2:** Logistic regression analysis for environment risks and miRNAs interaction effects.

	**β-estimate**	**SE**	***p*-value**	**OR**	**95% CI for OR**
hsa-miR-181b-5p	−6.41	1.08	<0.001	0.002	0–0.014
hsa-miR-320a	−7.50	1.12	<0.001	0.001	0–0.005
Family psychiatric history × hsa-miR-181b-5p	2.16	0.69	0.002	8.65	2.23–33.58
Neonatal jaundice × hsa-miR-19b-3p	0.89	0.23	<0.001	2.44	1.55–3.83
Maternal stress during pregnancy × hsa-miR-320a	3.67	0.95	<0.001	39.42	6.07–255.84

## Discussion

In this study, we enrolled 318 ASD and healthy children to explore the main effects of miRNAs as well as their interaction effects with well-replicated ASD environmental risk factors on the risk of ASD. We find that the dysregulation of hsa-miR-181b-5p, hsa-miR-320a, and hsa-miR-19b-3p could interact with environmental factors to impact the risk of ASD.

Previous studies demonstrate that ASD is a heritable disorder involving multiple gene networks (5). MicroRNAs could influence gene expression, playing important roles in neurodevelopment. It is reported that miRNAs could influence neurogenesis and synaptogenesis and participate in ASD pathogenesis, serving as the biomarkers of ASD (5). MicroRNAs in salivary or serum show high accuracies to differentiate control and ASD subjects (18, 19). Several miRNAs, including miR-181b-5p, miR-320a, miR-19b-3p, miR-106b, miR-140, and miR-199b are regarded as candidates to identify ASD (5). In the study conducted by Mundalil et al. (19), miR-181b-5p and miR-320a were downregulated, and miR-19b-3p was upregulated in ASD patients compared with controls. Due to the results of Mundalil's study, miR-181b-5p, miR-320a, and miR-19b-3p were chosen in this study to detect the interaction effects of miRNAs and well-replicated ASD environmental risk factors on the risk of ASD. Our results are in line with Mundalil's findings. We find that the serum levels of hsa-miR-181b-5p and hsa-miR-320a in ASD children were much lower than those in healthy controls, and the serum levels of hsa-miR-19b-3p in ASD children were much higher. The possible molecular mechanisms to underly miRNA upregulation or downregulation in ASD are still being investigated. A review published previously reveals that the location of specific miRNAs at copy number variant (CNV) loci in ASD may lead to their dysregulation (5). Another potential mechanism is that individual miRNA sequences are altered in ASD children (20).

Previously investigated environmental risk factors for ASD include paternal and maternal age, fetal environment, perinatal and obstetric events (e.g., hypoxia), smoking and alcohol use, nutrition, and toxic exposures (21). In this study, we find that the ASD group had significantly higher rates of maternal stress during pregnancy, maternal drinking during pregnancy, threatened abortion, pregnancy-induced hypertension, gestational diabetes, maternal anemia during pregnancy, umbilical cord knot, neonatal jaundice, family psychiatric history, and much lower birth weight compared with the control group. Parental or maternal age is a well-established risk factor for ASD (21). Unfortunately, in the present study, parental, and maternal age were not significantly different between the two groups. Lacking a large enough sample size may contribute to this result.

Gene–environment interaction is an emerging hypothesis to expound the increased incidence of ASD (22). MicroRNAs may be one of the factors to explain the gene–environment interaction. Hicks et al. (2) find that salivary miRNAs were associated with environmental factors to affect the risk of ASD. Nakata et al. (23) identified that the downregulation of miR-6126 was correlated with the severity of social deficits in ASD patients. In our study, we find that the dysregulation of hsa-miR-181b-5p, hsa-miR-320a, and hsa-miR-19b-3p could interact with environmental factors, such as maternal stress during pregnancy, neonatal jaundice, and family psychiatric history to impact the risk of ASD.

Maternal stress during pregnancy susceptibility appears to affect offspring neurodevelopment (24). The extent of this risk for ASD is investigated in a number of studies (25). Previously, maternal stress exposure was found to be associated with dysregulation of miRNAs in offspring brain in rats (26). This result indicates that miRNAs might be associated with maternal stress exposure, contributing to the ASD risk. As expected, we find that hsa-miR-320a could interact with maternal stress exposure to affect ASD risk in this study. The OR of this interaction is as high as 39.42. It is reported that total bilirubin levels can result in infants' neuronal injury (27). A recently published meta-analysis shows that neonatal jaundice is a potential risk factor for ASD (28). The results of this study confirm that the interaction of neonatal jaundice and hsa-miR-19b-3p might increase the risk of ASD. Children with a family psychiatric history were more likely to be diagnosed with ASD. Interestingly, in this study, we find that family psychiatric history could interact with hsa-miR-181b-5p, playing a role in increasing ASD risk.

Previous studies (29) and our study show that miRNAs in both serum and saliva are dysregulated in patients with ASD, indicating that miRNAs are promising diagnostic and prognostic biomarkers for ASD. Whether miRNAs could be used to diagnose ASD has not reach a consensus (29). Thus, further studies are needed to identify if miRNAs could be used to diagnose ASD. Whether miRNAs could be used as therapeutic targets for ASD should also be investigated.

The present study has several limitations. First, this study had a relatively small sample size. Second, most of the patients and healthy controls were from a single hospital. Third, we only used qRT-PCR to analyze the differentially expressed miRNAs. Other methods are also needed to confirm our results. Fourth, the environmental and biochemical risk factors for ASD mentioned in this study were not comprehensive. Risk factors, such as prenatal viral infection, zinc deficiency, and oxidative stress (30, 31) should also be discussed in further studies. Furthermore, in this study, the target genes of hsa-miR-181b-5p, hsa-miR-320a, and hsa-miR-19b-3p were predicted based on previous studies (5). The role of other miRNAs should be also investigated. Finally, how miRNA affects ASD were not detected in this study either. Further studies are required to figure out this issue.

## Conclusion

Our study finds that the serum levels of hsa-miR-181b-5p and hsa-miR-320a in ASD children are much lower than those in healthy controls, and the serum levels of hsa-miR-19b-3p in ASD children were much higher. The dysregulation of hsa-miR-181b-5p, hsa-miR-320a, and hsa-miR-19b-3p could interact with environmental factors, such as maternal stress during pregnancy, neonatal jaundice, and family psychiatric history to impact the risk of ASD.

## Data Availability Statement

The original contributions presented in the study are included in the article/supplementary material, further inquiries can be directed to the corresponding author/s.

## Ethics Statement

This study was conducted with approval from the Ethics Committee of North China University of Science and Technology. The patients/participants provided their written informed consent to participate in this study.

## Author Contributions

LC and WD have made substantial contributions to conception and design. NX, JD, BX, and JM acquisition of data, analysis, and interpretation of data. LC, RY, and LW have been involved in drafting the manuscript and revising it critically for important intellectual content. FF have given final approval of the version to be published. All authors contributed to the article and approved the submitted version.

## Funding

This work was supported by Major Project of Medical Science of Hebei Provincial Commission of Health and Family Planning (No. 20180734).

## Conflict of Interest

The authors declare that the research was conducted in the absence of any commercial or financial relationships that could be construed as a potential conflict of interest.

## Publisher's Note

All claims expressed in this article are solely those of the authors and do not necessarily represent those of their affiliated organizations, or those of the publisher, the editors and the reviewers. Any product that may be evaluated in this article, or claim that may be made by its manufacturer, is not guaranteed or endorsed by the publisher.
